# Association Between Sedentary Behavior and the Development of New-Onset Chronic Pain in Older American Adults: Insights From the Health and Retirement Study

**DOI:** 10.1177/08901171251412951

**Published:** 2025-12-28

**Authors:** Wilson Fandino, Karla Loureiro Loss, Nuno V. Gomes, José Armando García Delgado, Julia Daudt de Faro Salamonde, Blanca Bazán-Perkins, Kevin Pacheco-Barrios, Felipe Fregni

**Affiliations:** 1 Anaesthetics Department, 8945 Guy’s and St Thomas’ NHS Foundation Trust, London, UK; 2 Pediatrics Department, 28126 Federal University of Espirito Santo, Vitoria, Brazil; 3 Anesthesiology, 30262 University Hospital Basel, Basel, Switzerland; 4 Department of Clinical Research, University of Basel, Basel, Switzerland; 5 Department of Anaesthesia and Intensive Care, Royal Papworth Hospital NHS Foundation Trust, Cambridge, UK; 6 Hospitalization Department, Instituto Nacional Del Cáncer Rosa Emilia Sánchez de T., INCART, Santo Domingo, Dominican Republic; 7 Faculdade de Medicina São Leopoldo Mandic, SLMandic, Campinas, Brazil; 8 Laboratorio de Inmunofarmacología, 42635 Instituto Nacional de Enfermedades Respiratorias Ismael Cosio Villegas, Mexico City, Mexico; 9 Departament of Basic Sciences, Tecnológico de Monterrey, Escuela de Medicina y Ciencias de La Salud, Mexico City, Mexico; 10 Neuromodulation Center and Center for Clinical Research Learning, Spaulding Rehabilitation Hospital and Massachusetts General Hospital, Harvard Medical School, Boston, MA, USA

**Keywords:** chronic pain, sedentary behavior, obesity, preventive medicine

## Abstract

**Purpose:**

Explore the causal relationship between sedentary behavior (SB) and the new onset of chronic pain (NOCP) in Americans aged ≥50 years.

**Design:**

Retrospective secondary data analysis.

**Setting/Sample:**

In a target trial emulation framework, a dataset comprising four consecutive waves (2012, 2014, 2016, and 2018) from the longitudinal Health and Retirement Study was investigated.

**Measures/Analysis:**

Univariable and multivariable analyses were conducted using survey-weighted, mixed-effects Poisson regression models with one-level random intercepts and linearized standard errors. Using the same approach, causal mediation analyses were carried out to examine the potential role of obesity, depression, and sleep disturbances as mediators in the relationship between SB and NOCP. The role of education level, race, and sex as potential effect modifiers was also evaluated.

**Results:**

In the multivariable analysis, the risk of NOCP in participants who reported not having pain in the 2012 interview increased by 22% in those with SB vs those reporting no SB (95% CI = 1.12, 1.33; *P* < .001). The mediation effect attributed to obesity and depression was 58.2% (95% CI = 50.5, 71.3; *P* < .001) and 8.8% (95% CI = 1.6, 14.1; *P* < .001), respectively. The risk of NOCP in participants reporting SB was increased by 24.8% in women vs men (incidence rate ratio 1.25; 95% CI = 1.02, 1.52; *P* = .03).

**Conclusion:**

There is strong evidence for an association between SB and NOCP in the American subpopulation of women aged ≥ 50 years. This effect seems to be mainly mediated by obesity.

## Purpose

In the U.S., chronic pain is a major public health issue affecting nearly 20% of all adults.^1^ It represents up to $300 billion and $335 billion in direct and indirect costs per year, respectively.^2^ For the individual, chronic pain has physical, social and psychological implications, and is associated with higher levels of disability and accelerated cognitive decline, thus leading to a worse quality of life and premature death.^3-5^

Several risk factors, including age, sex, smoking, alcohol consumption, and comorbidities such as obesity, osteoarthritis, lung disease, stroke, and anxiety have been described as contributors for chronic pain.^6-10^ However, physical activity has been proven effective in providing relief.^11^ The benefits of exercise for chronic pain are well-known and supported by a large body of literature.^12,13^ In addition, sedentary behavior (SB), defined as any waking activity performed in a sitting or reclining posture with an energy expenditure of ≤1.5 metabolic equivalents,^14^ has been associated with all‐cause mortality, reduced quality of life, increased hospitalization rates, obesity, and poor mental health.^15-19^

Previous research has shown that older adults are among the most sedentary groups, spending an average of 8.5-9.5 h daily in sedentary activities.^20,21^ In addition, the new onset of chronic pain (NOCP), defined as pain that persists for more than 3 months,^22^ seems to be more prevalent in women and is associated with obesity, lower level of education, and poor self-rated health.^23^ In this setting, SB may play a key role in the development of NOCP.^24^

However, whether SB is causally related to NOCP, the extent to which SB may contribute to NOCP in subjects without preexisting pain conditions, and whether this causal effect varies across sex, race, and education level remains unclear.^25^ While most previous studies have assessed the relationship between SB and pain using a cross-sectional design,^26^ there is a need for longitudinal studies to establish causality between SB and NOCP. Accordingly, the exact mechanisms underlying the association between SB and chronic pain, and the social determinants of this relationship, including education level, race, and sex-related effects, as well as the role of obesity, depression, and sleep disturbances as potential mediators is yet to be explored.

Therefore, we conducted a retrospective study including participants interviewed in the Health and Retirement Study (HRS)^27^ between 2012 and 2018 within the framework of a target trial emulation.^28^ The primary objective was to explore the causal relationship between SB and NOCP in Americans aged ≥ 50 years. The secondary objectives were (i) to conduct a mediation analysis to evaluate the total, direct and indirect effects of obesity, depression and sleep disturbances for the association between SB and NOCP, and (ii) to investigate the potential role of sex, race, and education level as potential effect modifiers for this relationship. We hypothesized that (i) SB is associated with the development of NOCP over time in this population, and (ii) obesity is a mediator of the association between SB and the likelihood of NOCP.

## Methods

### Design

We used data from the publicly available HRS,^27^ a longitudinal study involving older American adults conducted by the University of Michigan. The HRS survey included >37 000 participants living in the US, representing nearly 23 000 households. Participants were interviewed face-to-face or via telephone by trained interviewers across the US.

In a target trial emulation framework, a dataset comprising four consecutive waves (2012, 2014, 2016, and 2018) was used to investigate the relationship between SB and NOCP among those who reported no pain in the 2012 wave. Information on data cleaning methodology is available in the Appendix Material.

### Sample

Inclusion criteria included adults aged ≥50 years participating in the HRS with no chronic pain, defined as those who answered “no” to the question: “Are you often troubled with pain?” Participants with missing pain status and unmatched participants after merging data from the 2012-2018 waves were excluded.

### Measures

#### Exposure

Information on SB was collected from the following questions included in Section C of the HRS questionnaire:^27^

(1) Moderate activity: “How often do you take part in sports or activities that are moderately energetic? These activities included gardening, cleaning the car, walking at a moderate pace, dancing, floor or stretching exercises.”
(2) Vigorous activity: “How often do you take part in sports or activities that are vigorous? These activities included jogging, running, swimming, cycling, aerobics or gym workout, playing tennis, or digging with a spade or shovel.”

Based on this information, a previously validated and similarly applied Physical Activity Index Score (PAIS) was generated.^29-31^ Specifically, moderate activity responses were coded as hardly ever (0), one to three times a month (1), once a week (3), or more than once a week (6).

Vigorous activity responses were coded as hardly ever (0), one to three times a month (2), once a week (6), or more than once a week (12). The generation of PAIS did not account for the available information on mild activity.^31^

Accordingly, the PAIS was calculated by summing the scores obtained for each question, ranging from zero (minimal activity) to 18 (maximal activity). This score was dichotomized using a cut-off of 6. Thus, individuals with a score of <6 were deemed to exhibit SB, unlike those with higher scores. In the longitudinal analysis, SB was considered persistent if it was exhibited by patients over consecutive years during the 2012 and 2014 waves.

#### Outcome

The outcome of interest was NOCP. Following the question, “Are you often troubled with pain?”, respondents were given five options: “Yes,” “No,” “Not ascertained,” “Refuse to answer,” and “Not applicable.” This variable was dichotomized as “Yes” or “No,” thus excluding from the analysis participants who provided a different answer.

#### Covariates

After drawing a direct acyclic graph (DAG) (Appendix Figure 1), the following covariates were considered potential confounders for the relationship between SB and NOCP: age (at the time of the 2012 wave), sex, race, education level, smoking, alcohol intake, and comorbidities including stroke, heart disease, diabetes, lung disease, mental disease, and cancer (excluding skin) codified as binary (yes/no). In addition, the variables sex, race, and educational level were analyzed as potential effect modifiers, and depression, sleep disturbances and obesity (defined as body mass index [BMI] >30) were considered potential mediators.

All analyses were conducted using STATA (version 18.5).

### Analysis

#### Descriptive Statistics

Categorical variables were described as frequencies and percentages according to SB status. The demographic characteristics of the sample were reported in terms of unweighted frequency, percentages, and weighted estimated proportions, based on an estimated target population. The distribution of covariates conditioned on SB status was explored, and the prevalence of missing data was obtained for all covariates.

#### Inferential Statistics

A cohort of survey respondents who reported being free of pain in the 2012 interview was retrospectively established. These participants were then followed for three consecutive waves (2014, 2016, and 2018) to investigate the development of NOCP. Univariable analyses using separate survey-weighted, mixed-effects Poisson (SWMEP) regression models were conducted to explore the crude association between each covariate and NOCP. All SWMEP regression models were carried out with one-level random intercepts and linearized standard errors.^32^ In addition, these models were constructed separately for each time-varying covariate (namely SB, smoking, alcohol intake, stroke, heart disease, diabetes, lung disease, mental disease, and cancer) and each wave. The estimates were represented in a forest plot to illustrate incidence rate ratio (IRR) changes over time and evaluate the need for modeling random slopes. Subsequently, a multivariable analysis using a SWMEP regression model was conducted. Along with SB, the following covariates were included in the saturated model: age, sex, race, education level, smoking, alcohol intake, stroke, heart disease, diabetes, lung disease, mental disease, and cancer (excluding skin). The obtained IRR for the multivariable analysis was interpreted as an approximation of a risk ratio.^32^

#### Sensitivity Analysis

Three sensitivity analyses were conducted to evaluate the consistency of results. In the first, obesity was deemed a confounder rather than a mediator and, therefore, included in the multivariable analysis. In the second, two multivariable SWMEP models were undertaken separately by fixing the exposure to one wave (2012) and three consecutive waves (2012, 2014, and 2016). The estimates obtained from these models were then compared with the SWMEP reported for a fixed exposure in two waves (2012 and 2014). In the third, a SWMEP model was employed using the information on time-varying covariates from the 2010 wave, while keeping the exposure fixed at two consecutive waves (2012 and 2014). The estimates obtained from this model were compared with those of the reported SWMEP model, which used information on time-varying covariates from the 2012 wave.

#### Causal Mediation and Effect Modification

The methodology used for causal mediation analysis^33-36^ is described in the Appendix Material and summarized in Appendix Figure 2. In short, our mediation analysis aimed to decompose the effects of obesity, depression and sleep disturbances for the relationship between SB and NOCP into total, direct and indirect effects. In addition, the covariates sex, race, and education level were separately incorporated in the multivariable SWMEP model to evaluate their potential as effect modifiers. The obtained estimates were reported as IRR with their corresponding *P*-values and 95% confidence intervals (CI), which were estimated with bootstrapping.

## Results

**Figure 1.**
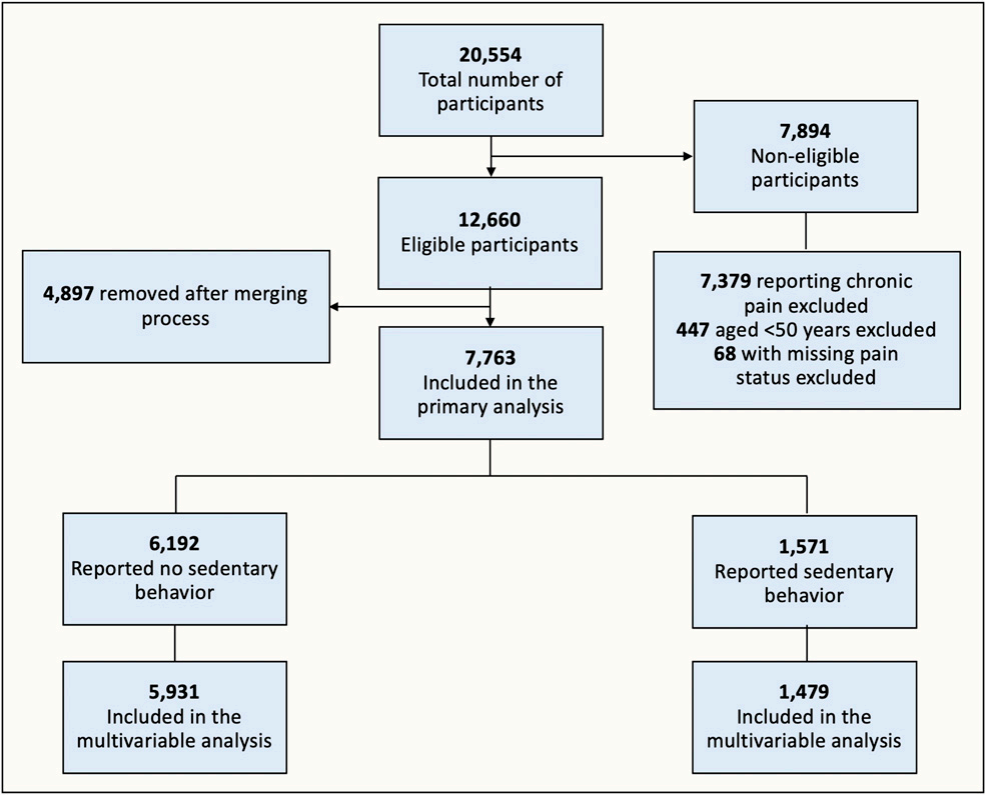
Flowchart representing eligible participants included in the study, in the framework of target trial emulation

From a total of 20 554 participants interviewed in 2012, 12 660 were eligible, of which 7763 (61.3%) were included in the study (Figure 1). Among them, 6192 (79.8%) reported no SB, and 1571 (20.2%) reported SB. The number of participants included in the multivariable analysis was 5931 (95.8%) and 1479 (93.7%), respectively.

The baseline demographic and health-related characteristics of the participants and the proportion of participants stratified by SB are presented in Table 1. The sample-weighted estimated proportion of females in the target population was 53.8%, whereas 41.0% were aged 50-60, 35.7% were aged 61-70, and 23.3% were >70 years. An estimated population proportion of 17.6% of participants was classified as exhibiting SB. The proportion of missing data for all observations was <2.3% (Appendix Figure 2). The variables SB, age and sex did not have missing observations.

**Table 1.** Demographic Characteristics for Respondents of the 2012 Wave With No Chronic Pain

Variable	Unweighted n (%)	Sedentary behavior n (%)		Weighted proportion (%)^a^	95% CI^b^
		No	Yes		
Sedentary behavior					
No	6192 (79.8)	-	-	82.4	80.9-83.8
Yes	1571 (20.2)	-	-	17.6	16.2-19.1
Age (years)					
50 to 60	2852 (36.7)	2370 (38.3)	482 (30.7)	41.0	38.2-43.9
61 to 70	2302 (29.7)	1856 (30.0)	446 (28.4)	35.7	33.9-37.6
>70	2609 (33.6)	1966 (31.8)	643 (40.9)	23.3	21.3-25.3
Sex					
Male	3331 (42.9)	3425 (55.3)	1007 (64.1)	46.2	52.6-54.9
Female	4432 (57.1)	2767 (44.7)	564 (35.9)	53.8	52.6-54.9
Race					
White/caucasian	5614 (72.5)	4553 (73.6)	1071 (68.3)	84.3	83.1-85.4
Black/African American	1506 (19.5)	1132 (18.3)	374 (23.9)	9.7	8.7-10.9
Other	622 (8.0)	499 (8.1)	123 (7.8)	6.0	5.2-6.8
Education level					
None	1138 (14.8)	836 (13.6)	302 (19.4)	9.6	8.3-11.0
High school	3897 (50.6)	3012 (49.0)	885 (56.8)	48.2	46.3-50.1
College	1704 (22.1)	1438 (23.4)	266 (17.1)	26.2	24.7-27.8
Master/professional	967 (12.6)	861 (14.0)	106 (6.8)	16.0	14.5-17.6
Smoking					
No	3733 (48.1)	3016 (48.7)	717 (45.6)	48.5	47.0-50.1
Yes	4029 (51.9)	3175 (51.3)	854 (54.4)	51.5	49.9-53.0
Alcohol intake					
No	3078 (39.7)	2201 (36.8)	797 (50.7)	34.9	32.8-37.1
Yes	4684 (60.4)	3910 (63.2)	774 (49.3)	65.1	62.9-67.2
Stroke					
No	7381 (95.5)	5923 (96.0)	1458 (93.6)	96.6	96.1-97.0
Yes	345 (4.5)	245 (4.0)	100 (6.4)	3.4	3.0-3.9
Heart disease					
No	6368 (82.7)	5142 (83.6)	1226 (78.9)	84.1	83.0-85.2
Yes	1337 (17.4)	1010 (16.4)	327 (21.1)	15.9	14.8-17.0
Diabetes					
No	6117 (79.5)	4999 (81.4)	1118 (72.1)	81.9	80.9-82.9
Yes	1579 (20.5)	1146 (18.7)	433 (27.9)	18.1	17.1-19.1
Lung disease					
No	7297 (94.4)	5869 (95.2)	1428 (91.4)	94.8	94.0-95.4
Yes	431 (5.6)	296 (4.8)	135 (8.6)	5.2	4.5-6.0
Mental disease					
No	6800 (88.3)	5468 (88.9)	1332 (85.6)	87.6	86.8-88.4
Yes	904 (11.7)	680 (11.1)	224 (14.4)	12.4	11.6-13.1
Cancer (excluding skin)					
No	6791 (87.7)	5425 (87.8)	1366 (87.3)	88.5	87.6-89.4
Yes	953 (12.3)	754 (12.2)	199 (12.7)	11.5	10.6-12.4
Obesity					
No	5155 (68.0)	4293 (70.8)	862 (56.5)	69.1	67.8-70.5
Yes	2431 (32.1)	1768 (29.2)	663 (43.5)	30.9	29.5-32.2
Depression					
No	6613 (85.3)	5328 (86.1)	1285 (81.9)	84.4	83.2-85.4
Yes	1143 (14.7)	859 (13.9)	284 (18.1)	15.6	14.6-16.8
Sleep disturbances^c^					
No	6589 (84.9)	5265 (85.1)	1324 (84.3)	84.1	82.9-85.1
Yes	1172 (15.1)	925 (14.9)	247 (15.7)	15.9	14.9-17.1

CI, confidence interval.

^a^Accounting for estimated population values. For a total sample size of 7763, the estimated population size was 37,878,092 community-dwelling American adults, aged ≥50 years, with no chronic pain.

^b^Estimated with linearized standard errors.

^c^Sleep medications used in the past 2 weeks.

In the univariable analysis, SB was associated with a 36.6% increased risk of NOCP (95% CI = 1.26,1.49; *P* < .001). Similarly, older age, smoking, heart disease, lung disease, mental disease, obesity, depression, sleep disturbances, stroke, and diabetes were associated with an increased risk of NOCP. In contrast, males, practicing moderate or vigorous exercise more than once a week, higher education level, and not abstaining from alcohol were associated with a decreased risk of NOCP (Table 2).

**Table 2.** Univariable and Multivariable Analyses Based on Survey-Weighted Poisson Regression Models for the 2018 Wave

Variable	Univariable analysis					Multivariable analysis (n = 7,410^a^, n = 28 746^b^)		
	*n* ^a^	*n* ^b^	IRR	95% CI	*P* value	IRR	95% CI	*P* value
Sedentary behavior^c,d^	7750	30 042	1.37	1.26-1.49	<.001	1.22	1.12-1.33	**<.001**
Age in 2012 (years)	7750	3042						
50 to 60	-	-	-	-	-	-	-	-
61 to 70	-	-	1.15	1.03-1.28	.011	1.08	0.97-1.20	.164
>70	-	-	1.15	1.05-1.26	.002	1.02	0.93-1.12	.642
Race	7729	29 966						
White/Caucasian	-	-	-	-	-	-	-	-
Black/African American	-	-	1.09	0.96-1.23	.176	1.03	0.90-1.17	.713
Other	-	-	0.95	0.82-1.09	.436	0.90	0.76-1.07	.217
Education level	7693	29 834						
None	-	-	-	-	-	-	-	-
High school	-	-	0.89	0.80-0.98	.029	0.91	0.81-1.02	.097
College	-	-	0.77	0.68-0.88	<.001	0.84	0.73-0.97	**.017**
Master/professional	-	-	0.72	0.62-0.83	<.001	0.80	0.70-0.92	**.002**
Sex (0 = male, 1 = female)	7750	30 042	1.18	1.09-1.30	<.001	1.14	1.03-1.23	**.010**
Smoking^d^	7749	30 038	1.09	1.01-1.19	.037	1.08	0.98-1.19	.114
Alcohol intake^d^	7749	30 038	0.80	0.75-0.86	<.001	0.90	0.82-0.99	**.032**
Stroke^d^	7713	29 894	1.29	1.05-1.58	.016	1.09	0.87-1.37	.425
Heart disease^d^	7692	29 826	1.22	1.09-1.36	.001	1.11	0.99-1.25	.078
Diabetes^d^	7684	29 791	1.27	1.16-1.40	<.001	1.17	1.07-1.29	**.001**
Lung disease^d^	7715	29 907	1.44	1.25-1.65	<.001	1.19	1.02-1.37	**.023**
Mental disease^d^	7692	29 812	1.64	1.46-1.85	<.001	1.55	1.36-1.78	**<.001**
Cancer (excluding skin)^d^	7731	29 966	1.09	0.96-1.24	.199	1.04	0.92-1.17	.534
Obesity^d^	7573	29 364	1.42	1.30-1.55	<.001	-	-	-
Depression^d^	7743	30 014	1.58	1.42-1.75	<.001	-	-	-
Sleep disturbances^d,e^	7748	30 034	1.44	1.27-1.62	<.001	-	-	-
PAIS^f^	7721	29 883	0.96	0.96-0.97	<.001	-	-	-
Moderate physical activity^d,g^	7739	29 978	0.74	0.69-0.79	<.001	-	-	-
Vigorous physical activity^d,g^	7727	29 924	0.65	0.59-0.71	<.001	-	-	-

PAIS, physical activity index score.

Boldface indicates statistical significance (*P* < .05).

^a^Number of participants interviewed in 2012.

^b^Number of observations collected across 4 waves (2102, 2014, 2016, and 2018).

^c^Defined as no (PAIS<6) or yes (PAIS ≥ 6).

^d^Variables codified as 0 = no and 1 = yes.

^e^Sleep medications used in the past 2 weeks.

^f^PAIS ranging from 0 (no physical activity) to 18 (vigorous physical activity).

^g^Defined as more than once a week.

**Figure 2.**
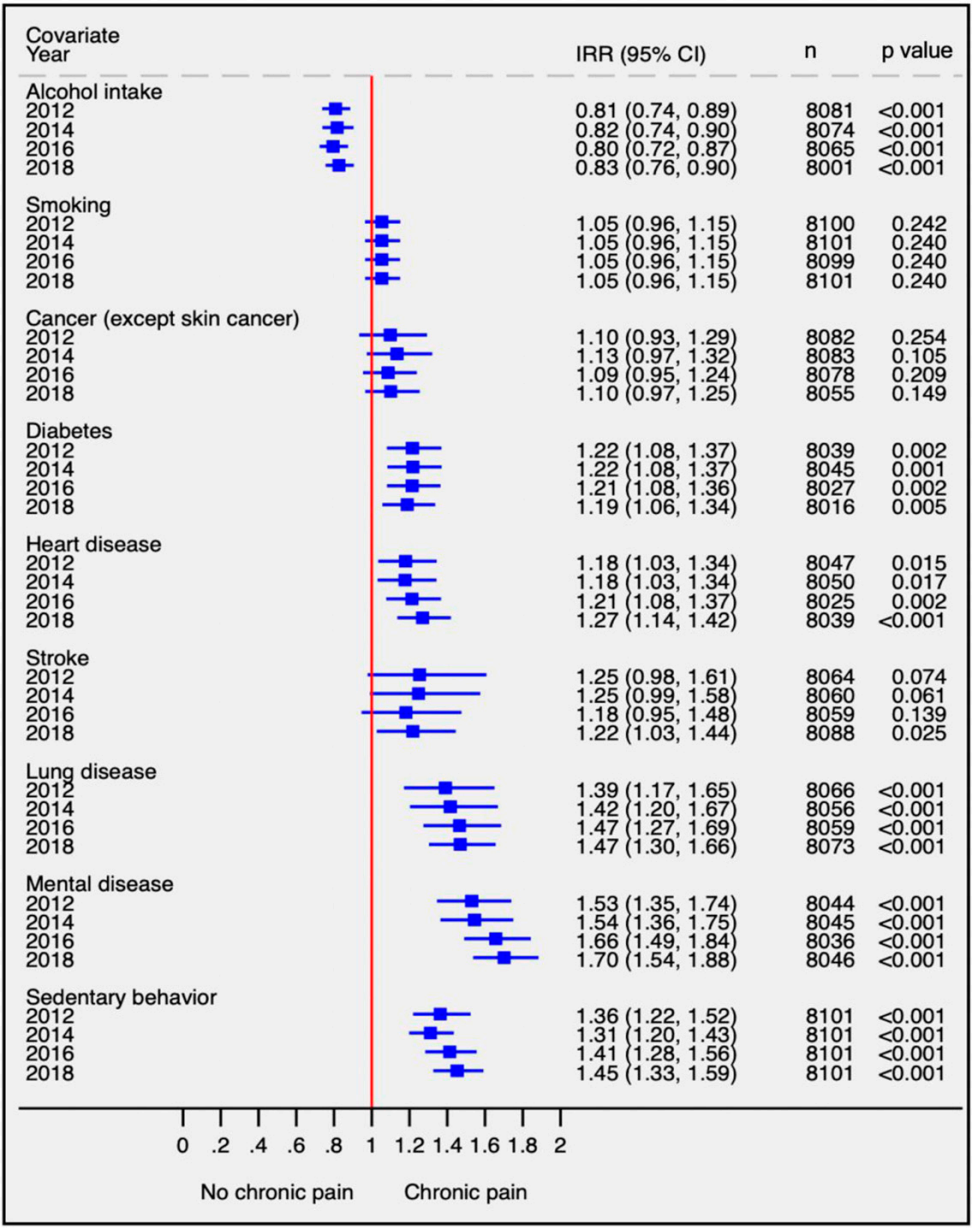
Forest plot exploring the crude association between each covariate and the new onset of pain. The survey-weighted univariable analyses obtained for 2012, 2014, 2016, and 2018 waves are compared with respect to the new onset of pain reported in 2018. All covariates are binary (0 = no, 1 = yes). IRR, incidence rate ratio; n, number of participants

When conducting separated univariable analyses to explore the association between SB and NOCP for each covariate across four waves (2012, 2014, 2016, and 2018), there were no consistent changes in the trends of the estimates for any of the covariates (Figure 2).

A DAG for the relationship between SB and NOCP is provided in Appendix Figure 1. Accordingly, the multivariable model included all the above-mentioned covariates as potential confounders. After adjusting for these covariates, the risk of NOCP in participants free of pain in 2012 increased by 22% in those with SB vs those reporting no SB (95% CI = 1.12, 1.33; *P* < .001) (Table 2). In exploring the effect of SB as compared to moderate or vigorous exercise in the multivariable analysis, after adjusting for the same covariates, we found that the risk increased by 15% for participants reporting moderate exercise (95% CI = 1.05-1.26, *P* = .001), whereas the risk increased by 17% among participants reporting vigorous exercise (95% CI = 1.07-1.27, *P* = .004) (Appendix Table 1).

### Sensitivity Analysis

A description of all sensitivity analyses conducted is provided in Appendix Tables 2-4. When adding obesity, depression, and sleep disturbances as potential confounders to the multivariable model, the estimated increased risk of NOCP in participants with SB did not change considerably (IRR = 1.16; 95% CI = 1.06,1.28; *P* = .002) (Appendix Table 2). When fixing SB to one wave (2012) or three waves (2012, 2014, and 2016), the obtained estimates for the association between SB and NOCP were consistent with the multivariable model described in Appendix Table 3. When information on time-varying covariates (ie, smoking, alcohol intake, stroke, heart disease, diabetes, lung disease, mental disease, and cancer) were obtained from the 2010 wave,^35^ similar results were obtained for an increased risk of NOCP (IRR = 1.26; 95% CI = 1.15,1.37; *P* < .001) (Appendix Table 4).

### Causal Mediation

We used the same SWMEP model structure for our mediation analysis. The proportion attributed to obesity for the estimated effect of SB on NOCP was 58.2% (95% CI = 50.5,71.3; *P* < .001); the proportion attributed to depression and sleep disturbances was 8.8% (95% CI = 1.6,14.1; *P* < .001) and 7.3% (95% CI = −3.37,14.53; *P* = .117), respectively (Appendix Figure 3).

### Effect Modification

The risk of NOCP in participants reporting SB was increased by 24.8% in women vs men (IRR = 1.25; 95% CI = 1.02,1.52; *P* = .030). In a subgroup analysis, the risk of NOCP in women reporting SB was increased by 33.1% (IRR = 1.33; 95% CI = 1.19, 1.48; *P* < .001) compared to women not reporting SB. By contrast, the risk of NOCP in men reporting SB did not increase significantly (IRR = 1.05; 95% CI = 0.89, 1.23; *P* = .57) compared to men not reporting SB (Appendix Table 5 and Appendix Figure 4). Race and educational level did not substantially modify the relationship between SB and NOCP (Appendix Table 5).

## Discussion

Our results provide strong evidence for an increased risk of NOCP in American women aged ≥50 years reporting SB, primarily mediated by obesity. In line with previous studies,^31^ participants aged between 50 and 60 years were also included, as the age category level of reference to highlight the need for introducing behavioral changes as early in life to prevent NOCP. For example, in a previous study involving >33 000 participants aged 37-73 years, authors reported a 33% increase in new-onset low back pain. However, when stratified by sex, men were at a higher risk (RR = 1.38; 95% CI = 1.12, 1.69) compared to women (RR = 1.29; 95% CI = 1.08,1.54).^36^ Another study involving 59 office workers showed that prolonged sitting (>4.5 h) was associated with an increase in back muscle stiffness, which could contribute to the development of low-back pain.^37^

We hypothesized that the effect of SB in NOCP could vary across sex, race, and education level. In our study, sex was a strong effect modifier, thus making sedentary women more prone to developing NOCP. Our findings also indicate that these mediation effects are particularly pronounced in women. One plausible explanation is that SB fosters a state of chronic low-grade inflammation, which in turn may lead to or exacerbate both obesity and depression. Although inflammation was not directly measured in our study, the biological plausibility of this mediator is supported by existing studies linking sedentary lifestyles to elevated inflammatory markers.^38^ As women often exhibit a more pronounced inflammatory response, this could partly explain the stronger association between SB, obesity, depression, and pain observed in female populations.^39,40^ Another study reported a higher prevalence of chronic pain in women, particularly owing to hormonal influences, differential brain activation, and variations in the endogenous opioid system.^41^ In previous studies, we demonstrated the important disruptive effect of obesity on pain control.^42,43^ Future research should, therefore, aim to elucidate the role of inflammation as a critical intermediary factor in the complex interplay among sedentary behavior, obesity, depression, and pain, potentially offering novel insights into sex-specific intervention strategies. In contrast, no evidence of an effect modification from race or education level was found.

Our study showed that 58.2% of the effect of SB on NOCP was mediated by obesity. Although the precise mechanisms linking obesity, SB, and pain are not fully understood, several biological pathways may explain this relationship. Previous research has proposed SB as a key factor in increasing abdominal obesity, BMI and the risk of obesity.^44-46^ In addition, obesity has been associated with both greater pain severity and pain interference, as well as with SB.^19,47^ Both SB and obesity have been previously associated with higher levels of inflammatory markers such as interleukin-6, tumor necrosis factor-α, and C-reactive protein.^48-50^ These pro-inflammatory mediators can stimulate visceral nociceptors and sensitize nociceptive neurons in both the peripheral and central nervous systems. This mechanism reinforces the potential role of obesity as a causal mechanism for the relationship between SB and NOCP.^51,52^ However, whether obesity can be causally related to SB poses a different research question, which opens new avenues for further exploring this relationship in future studies.

In women with fibromyalgia, SB has been associated with altered pain modulation and brain regions involved in the sensory-discriminative brain areas.^53^ Additionally, obesity promotes a pathological interaction between adipose tissue and nociceptive afferent fibers, ultimately leading to increased pain perception.^54^ However, in a study including overweight middle-aged adults, standing time was associated with higher levels of pain-related disability, whereas longer sedentary behavior was not associated with pain disability.^55^

In our study, the proportion of the effect of SB on NOCP attributed to depression was 8.8%. The relationship between SB and depression was previously explored in the Irish Longitudinal Study on Ageing, which highlighted the influence of physical inactivity on the relationship between SB and depression in adults aged ≥50 years.^56^ Another study exploring genetic polymorphisms associated with chronic pain found an association between (i) chronic pain and depression and (ii) chronic pain and SB, but not between SB and depression, thus emphasizing the complex relationship among these three factors and the importance of assessing the potential mechanisms involved.^57^

Our results show that SB is strongly associated with increased pain and suggest that this relationship is partly mediated by obesity and depression. While these mediation effects are biologically plausible, obesity and depression could also be potential confounders for the association between SB and NOCP. In this scenario, some authors recommend that this variable should be excluded from multivariable analysis to prevent bias.^34^ When the variables obesity, depression and sleep disturbances were included as confounders in the sensitivity analysis, the results remained unchanged, thus providing consistency of our results.

The results presented in our mediation analysis suggest that designing public health strategies aimed at reducing the prevalence of obesity may have an impact on the overall reduction of the causal effect of SB on NOCP. Nevertheless, policymakers should recognize that obesity may not be easily reversible through conventional interventions.

The present study had several strengths. First, the large sample size allowed us to explore a subpopulation of subjects with no pain. This strategy was used in the context of a target trial emulation to understand the role of SB in this subpopulation. Second, the amount of missing data was low (<2.3% for all covariates) as compared to similar studies involving publicly available datasets.^58^ Third, the use of a strong methodological strategy and the consistency of sensitivity analyses suggest the reliability of the results.^59^ Specifically, the use of multivariable Poisson regression models has been described as an alternative to logistic regression when dealing with binary outcomes.^60^ In our study, using logistic regression was not a good alternative, owing to lack of convergence of the model. Fourth, our study used longitudinal data unlike other studies involving cross-sectional data,^26^ thereby facilitating the causal interpretation of our findings. The longitudinal design enabled us to establish causality by not only estimating the magnitude of the effect of SB on NOCP, but also the temporality of this association.

### Limitations

Data collected in the HRS were not specifically designed to address our research question. This issue posed challenges related to the coding of variables, the generation of the variable SB as the exposure of interest, and the unmeasured confounders. For example, covariates capturing information on an unhealthy diet, a well-known contributor to chronic pain and systemic inflammation, were not available in the HRS dataset.

The variables pain, physical activity level, and all health-related data were self-reported and therefore susceptible to recall bias and misclassification. However, answering “yes” to the item assessing pain on the HRS questionnaire was previously shown to correspond to pain that at least moderately interferes with daily life,^61^ and previous research has shown a good correlation between self-reported and actual weight.^62^

The scores used for PAIS and SB definitions were previously used in studies involving the HRS population to assess physical activity levels.^29-31^ However, there is a need for unifying criteria of SB in future studies, as there is no consensus as to what sedentary activities are typical for an individual exhibiting SB. Thus, the results of our study may vary according to such definitions.

In addition, caution must be taken when interpreting the apparent protective effect of alcohol in participants reporting SB for the development of NOCP. First, the interpretation of estimates other than the one corresponding to the exposure of interest can be misleading.^63^ Second, the impact of studies involving large sample sizes on the statistical significance of results has been widely recognized.^64^ While the interpretation of results based on the statistical significance is discouraged,^65^ the clinical significance of our findings should guide policy makers to design strategies focused on promoting physical activity and reducing the prevalence of obesity, particularly in older women. However, since our study aimed to investigate the relationship between SB and NOCP, further research is needed to explore the potential causal effect of obesity on NOCP.

## Conclusions

There is strong evidence for an association between SB and NOCP in the American women, aged ≥50 years, who participated in the 2012 HRS interview. This effect seems to be mainly mediated by obesity. While the results of the present study are consistent and clinically relevant, more research is warranted to explore the role of SB in the development of NOCP in this population.

**So What?**

**What is Already Known About This Topic?**

While sedentary behavior (SB) has been linked to obesity, depression, and chronic pain, its impact on new onset of chronic pain (NOCP) remains unknown. In particular, the role of sex, race and educational level in this relationship is yet to be explored.

**What Does This Article Add?**

This study provides strong evidence for an increased risk of NOCP in American women aged ≥50 years who reported being sedentary, with obesity playing a key mediating role in this relationship. By identifying modifiable risk factors, this study supports the development of evidence-based interventions to reduce the burden of NOCP.

**What Are the Implications for Health Promotion Practice or Research?**

Further prospective research is warranted to explore the underlying mechanisms that could explain the link between SB and NOCP. From a health promotion perspective, there is a need for public health initiatives aimed at prioritizing programs to increase physical activity among older women, thereby minimizing the long-term consequences of SB and obesity.

## Supplemental Material

Supplemental Material - Association Between Sedentary Behavior and the Development of New-Onset Chronic Pain in Older American Adults: Insights From the Health and Retirement Study Supplemental Material for Association Between Sedentary Behavior and the Development of New-Onset Chronic Pain in Older American Adults: Insights From the Health and Retirement Study by Wilson Fandino, Karla Loureiro Loss, Nuno V. Gomes, José Armando García Delgado, Julia Daudt de Faro Salamonde, Blanca Bazán-Perkins, Kevin Pacheco-Barrios, and Felipe Fregni in American Journal of Health Promotion
