# Supplementary material for: Association Between Sedentary Behavior and the Development of New-Onset Chronic Pain in Older American Adults: Insights From the Health and Retirement Study
Source: Am J Health Promot. 2025 Dec 28;40(7):820–31. doi: 10.1177/08901171251412951 (PMC13365315; doi:10.1177/08901171251412951)

APPENDIX MATERIAL

Data Cleaning

The information on comorbidity status (i.e., stroke, heart disease, diabetes, lung disease, mental disease, and cancer), alcohol intake, smoking, and SB status, were obtained from the 2012 interview. In addition, the variables sex, age, education level, and race were obtained from tracked data available on the HRS website [link for tracked data]. Smoking status was dichotomized (yes/no) and included current and ex-smokers in the same category. The obesity variable was constructed from information on BMI, which was obtained from self-reported weight available in the 2012 wave, and height collected across five waves (2004, 2006, 2008, 2010, and 2012), since height was only available for first-time respondents. All covariates were categorized accordingly. The outcome of interest (NOCP) was obtained from the 2018 interview.

Causal Mediation

Let M represent the mediators (i.e., obesity, depression, and sleep disturbances), C the estimated total effect, C' the estimated direct effect, and AB the estimated indirect effect. The total effect (C) was decomposed using a three-stage strategy.[32] In the first stage, a SWMEP regression model (Model A) was applied to examine the relationship between SB and M. In the second stage, another SWMEP regression model (Model B) was used to quantify the association between M, considered the exposure in this context, and NOCP, the outcome. Thus, AB was estimated as the product of the coefficients obtained for Models A and B. In the last stage, an SWMEP regression model including SB and M as predictors and NOCP as the outcome was performed. This model was used to quantify C'. Finally, C was estimated as C'+AB, and the estimated mediation proportion attributed to M was measured as AB/C (Appendix Figure 2). The

95% confidence intervals of the estimated proportions were computed using bootstrapping after 1,000 replications.[33,34] An alternative saturated SWMEP including obesity, depression, and sleep disturbances was also conducted to evaluate the impact of these covariates as potential confounders.

Appendix Table 1. Multivariate analyses based on the survey-weighted Poisson regression models for the 2018 wave and comparing (i) moderate exercise vs. sedentary behavior, and (ii) vigorous exercise vs. sedentary behavior. The analysis included 7,410 participants interviewed in 2012 and 28,746 participants collected across four waves (2012, 2014, 2016, and 2018).

**Variable**	Moderate exercise[a]			Vigorous exercise[a]		
	**IRR**	**95% CI**	***p* value**	**IRR**	**95% CI**	***p* value**
Sedentary behavior[b,c]	1.15	1.05-1.26	**0.001**	1.17	1.07-1.27	**0.004**
Age in 2012 (years)						
50 to 60	-	-	-	-	-	-
61 to 70	1.08	0.97-1.20	0.160	1.08	0.97-1.20	0.160
>70	1.02	0.97-1.20	0.611	1.02	0.93-1.12	0.662
Race						
White/Caucasian	-	-	-	-	-	-
Black/African American	1.02	0.89-1.17	0.729	1.03	0.90-1.18	0.672
Other	0.90	0.76-1.06	0.206	0.89	0.75-1.06	0.197
Education level						
None	-	-	-	-	-	-
High school	0.91	0.82-1.02	0.104	0.90	0.81-1.01	0.071
College	0.84	0.73-0.97	**0.019**	0.84	0.73-0.96	**0.013**
Master/professional	0.80	0.70-0.92	**0.002**	0.79	0.69-0.91	**0.001**
Sex (0=male, 1=female)	1.14	1.04-1.25	**0.006**	1.12	1.02-1.23	**0.019**
Smoking[c]	1.08	0.98-1.19	0.107	1.08	0.98-1.18	0.126
Alcohol intake[c]	0.90	0.82-0.99	**0.028**	0.90	0.82-0.99	**0.033**
Stroke[c]	1.09	0.87-1.37	0.428	1.09	0.87-1.36	0.455
Heart disease[c]	1.11	0.99-1.25	0.067	1.10	0.98-1.24	0.091
Diabetes[c]	1.18	1.07-1.30	**0.001**	1.18	1.07-1.30	**0.002**
Lung disease[c]	1.19	1.03-1.38	**0.022**	1.19	1.03-1.38	**0.019**
Mental disease[c]	1.56	1.36-1.79	**<0.001**	1.56	1.36-1.79	**<0.001**
Cancer (excluding skin) [c]	1.04	0.92-1.18	0.512	1.04	0.92-1.17	0.564

[a] Defined as more than once a week.
[b] Defined as no (PAIS<6) or yes (PAIS≥6).
[c] Variables codified as 0=no and 1=yes.
PAIS: Physical activity index score, ranging from 0 (no physical activity) to 18 (vigorous physical activity).
Boldface indicates statistical significance (*p*<0.05).

Appendix Table 2. Sensitivity analysis for the association between sedentary behavior and new onset of chronic pain, after including obesity, depression, and sleep disturbances as potential confounders in the multivariate model presented in Table 2. The analysis included 7,245 participants interviewed in 2012 and 28,108 observations collected across 4 waves (2012, 2014, 2016, and 2018).

Variable	IRR	95% CI	*p* value
Sedentary behavior[a]	1.16	1.06-1.28	**0.002**
Age (years)			
50 to 60	-	-	-
61 to 70	1.11	1.00-1.23	**0.049**
>70	1.08	0.99-1.19	0.091
Race			
White/Caucasian	-	-	-
Black/African American	1.03	0.90-1.17	0.711
Other	0.89	0.74-1.03	0.189
Education level			
None	-	-	-
High school	0.86	0.77-0.97	**0.014**
College	0.79	0.69-0.91	**0.002**
Master/professional	0.77	0.66-0.91	**0.002**
Sex (0=male, 1=female)	1.09	1.00-1.19	0.063
Smoking[a]	1.07	0.97-1.18	0.162
Alcohol intake[a]	0.90	0.82-0.99	**0.033**
Stroke[a]	1.06	0.85-1.32	0.606
Heart disease[a]	1.08	0.97-1.21	0.170
Diabetes[a]	1.10	0.99-1.22	0.075
Lung disease[a]	1.14	0.99-1.31	0.078
Mental disease[a]	1.24	1.03-1.49	**0.023**
Cancer (excluding skin)[a]	1.04	0.92-1.17	0.576
Obesity[a]	1.37	1.25-1.50	**<0.001**
Depression[a]	1.27	1.10-1.46	**0.001**
Sleep disturbances[a,b]	1.32	1.15-1.51	**<0.001**

[a]Variables codified as 0=no and 1=yes.
[b] Sleep medications used in the past two weeks.
Boldface indicates statistical significance (*p*<0.05)

Appendix Table 3. Sensitivity analysis comparing three multivariate survey-weighted, mixed effects Poisson regression models (SWMEP) for the association between sedentary behavior and new onset of chronic pain. For all models, sedentary behavior has been defined as having a physical activity index score (PAIS) <6. In participants with PAIS ≥6, sedentary behavior was considered to be absent. Models 1, 2, and 3 were constructed based on the information obtained from a single wave (2012), two consecutive waves (2012 and 2014), and three consecutive waves (2012, 2014, and 2016), respectively.

	**Model 1 (*n*=7,798,[a] *n*=28,846[b])**			**Model 2 (*n*=7,410,[a] *n*=29,746[b])**			**Model 3 (*n*=7,763,[a] *n*=28,746[b])**		
**Variable**	**IRR**	**95% CI**	***p* value**	**IRR**	**95% CI**	***p* value**	**IRR**	**95% CI**	***p* value**
Sedentary behavior[c]	1.25	1.12-1.39	<0.001	1.22	1.12-1.33	<0.001	1.19	1.09-1.30	**<0.001**
Age (years)									
50 to 60	-	-	-	-	-	-	-	-	-
61 to 70	1.08	0.97-1.21	0.152	1.08	0.97-1.20	0.164	1.08	0.97-1.20	0.162
>70	1.03	0.93-1.13	0.607	1.02	0.93-1.12	0.642	1.02	0.93-1.12	0.615
Race									
White/Caucasian	-	-	-	-	-	-	-	-	-
Black/African American	1.02	0.89-1.17	0.756	1.03	0.90-1.17	0.713	1.03	0.90-1.18	0.687
Other	0.89	0.75-1.06	0.193	0.90	0.76-1.07	0.217	0.90	0.76-1.07	0.227
Education level									
None	-	-	-	-	-	-	-	-	-
High school	0.91	0.82-1.02	0.115	0.91	0.81-1.02	0.097	0.91	0.81-1.02	0.091
College	0.84	0.73-0.97	0.019	0.84	0.73-0.97	0.017	0.84	0.73-0.97	**0.016**
Master/professional	0.80	0.70-0.93	0.003	0.80	0.70-0.92	0.002	0.79	0.69-0.91	**0.002**
Sex (0=male, 1=female)	1.12	1.02-1.23	0.013	1.14	1.03-1.23	0.010	1.14	1.03-1.23	**0.010**
Smoking[c]	1.07	0.97-1.18	0.161	1.08	0.98-1.19	0.114	1.08	0.98-1.19	0.112
Alcohol intake[c]	0.91	0.83-1.00	0.046	0.90	0.82-0.99	0.032	0.90	0.82-0.99	**0.027**
Stroke[c]	1.08	0.86-1.35	0.502	1.09	0.87-1.37	0.425	1.10	0.87-1.38	0.424
Heart disease[c]	1.10	0.98-1.24	0.088	1.11	0.99-1.25	0.078	1.11	0.99-1.25	0.078
Diabetes[c]	1.16	1.05-1.28	0.003	1.17	1.07-1.29	0.001	1.18	1.08-1.30	**0.001**
Lung disease[c]	1.21	1.04-1.40	0.014	1.19	1.02-1.37	0.023	1.19	1.03-1.38	**0.020**
Mental disease[c]	1.55	1.36-1.78	<0.001	1.55	1.36-1.78	<0.001	1.55	1.36-1.78	**<0.001**
Cancer (excluding skin)[c]	1.03	0.92-1.17	0.593	1.04	0.92-1.17	0.534	1.04	0.92-1.17	0.546

[a] Number of participants interviewed in 2012.
[b] Number of observations.
[c] Variables codified as 0=no and 1=yes.
Boldface indicates statistical significance (*p*<0.05)

Appendix Table 4. Sensitivity analysis for the association between sedentary behavior and new onset of chronic pain. The analysis includes 7,608 participants and 28,536 observations. The information on sedentary behavior was obtained from the 2012 wave, whereas the time-varying covariates smoking, alcohol intake, stroke, heart disease, diabetes, lung disease, mental disease, and cancer (excluding skin) were obtained from the 2010 wave.

Variable	IRR	95% CI	*p* value
Sedentary behavior[a]	1.26	1.15-1.37	**<0.001**
Age (years)			
50 to 60	-	-	-
61 to 70	1.08	0.97-1.20	0.145
>70	1.02	0.93-1.12	0.677
Race			
White/Caucasian	-	-	-
Black/African American	1.04	0.91-1.19	0.540
Other	0.89	0.76-1.05	0.177
Education level			
None	-	-	-
High school	0.94	0.83-1.06	0.326
College	0.87	0.76-1.00	**0.047**
Master/professional	0.81	0.69-0.96	**0.015**
Sex (0=male, 1=female)	1.14	1.03-1.23	**0.009**
Smoking[a]	1.10	1.01-1.20	**0.033**
Alcohol intake[a]	0.94	0.86-1.03	0.169
Stroke[a]	1.09	0.83-1.43	0.521
Heart disease[a]	1.06	0.93-1.21	0.348
Diabetes[a]	1.16	1.05-1.29	**0.005**
Lung disease[a]	1.11	0.95-1.31	0.185
Mental disease[a]	1.49	1.30-1.71	**<0.001**
Cancer (excluding skin)[a]	1.07	0.93-1.23	0.343

[a]Variables codified as 0=no and 1=yes.
Boldface indicates statistical significance (p<0.05)

Appendix Table 5. Effect Modification of the Variables Sex, Race, and Education Level for the Relationship Between Sedentary Behavior and New Onset of Chronic Pain

Variable	IRR	95% CI	*p* value
Sex			
Male	-	-	-
Female	1.25	1.02-1.52	**0.030**
Race			
White/Caucasian	-	-	-
Black/African American	1.03	0.82-1.28	0.817
Other	0.93	0.58-1.48	0.761
ucation level			
None	-	-	-
High school	1.21	0.87-1.68	0.247
College	1.33	0.92-1.92	0.128
Master/professional	1.08	0.68-1.74	0.735

Boldface indicates statistical significance ($p<0.05$)

Appendix Figure 1. A proposed direct acyclic graph (DAG) for the relationship between sedentary behavior and new onset of chronic pain.

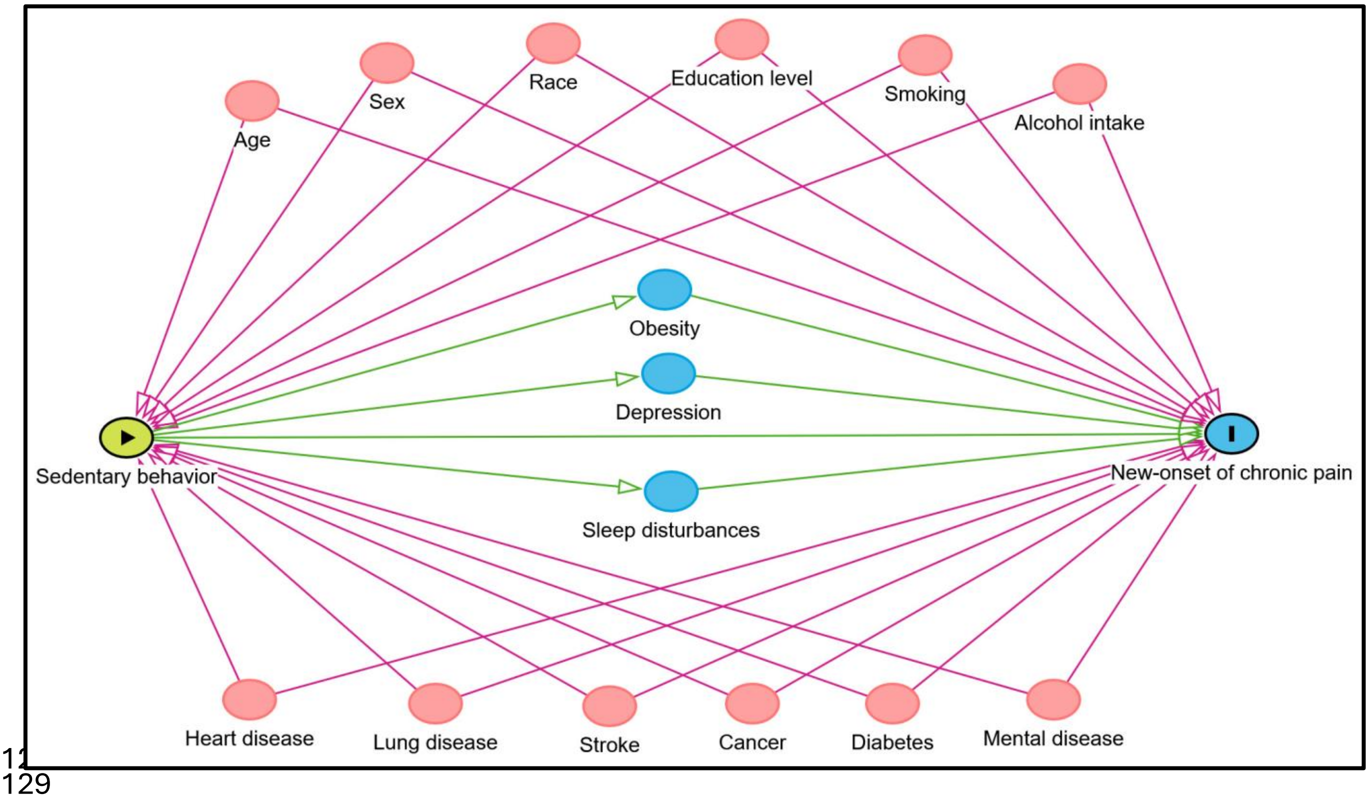

Appendix Figure 2. Number of missing observations obtained from the 2012 interview. A total of 68 participants with missing pain status were excluded from the study. The variables sedentary behavior, age, and sex did not have missing observations. Therefore, they are not included in this graph. *n*: number of respondents included in this study.

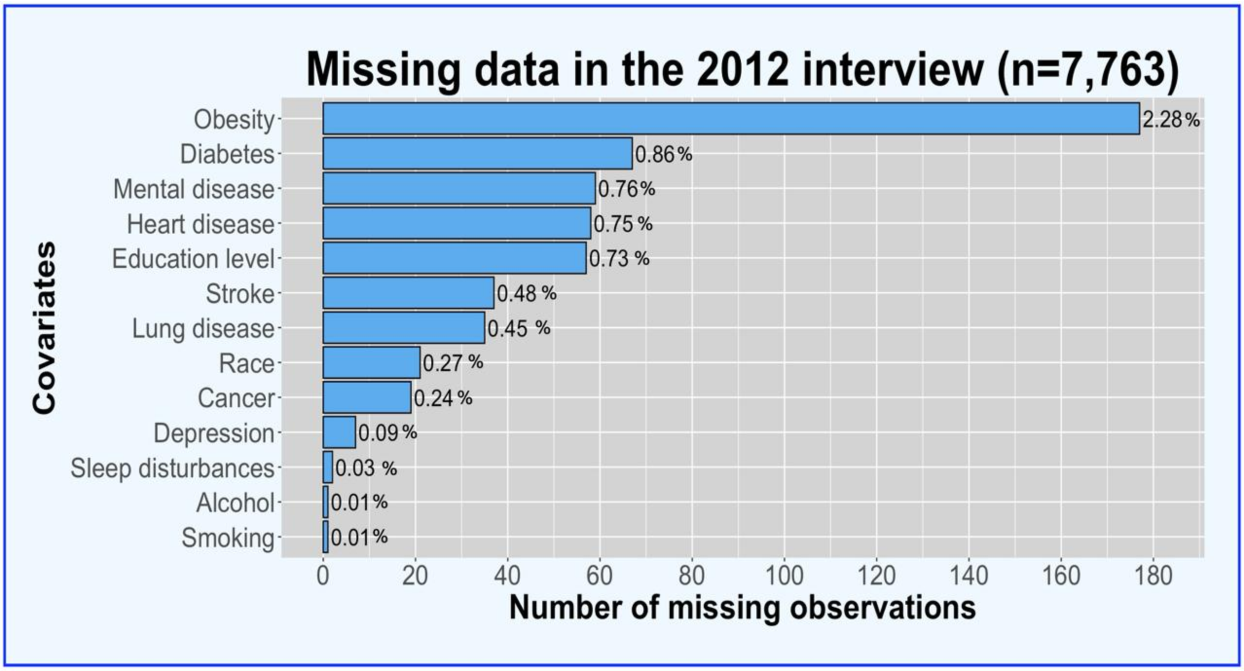

Appendix Figure 3. Direct acyclic graph (DAG) representing the effect of the mediators obesity, depression, and sleep disturbances (M) for the association between sedentary behavior (SB) and new onset of chronic pain (NOCP). A: Survey-weighted, mixed effects Poisson regression model with random intercepts (SWMEP) for the association between SB (exposure) and M (outcome). B: SWMEP for the association between M (exposure) and NOCP (outcome). C': SWMEP for the association between SB (exposure) and NOCP (outcome), adjusted for M. In this DAG, the indirect effect is represented by the product of the obtained estimates from A and B, and the direct effect is estimated by C'. Thus, the total effect is computed as C' + AB.

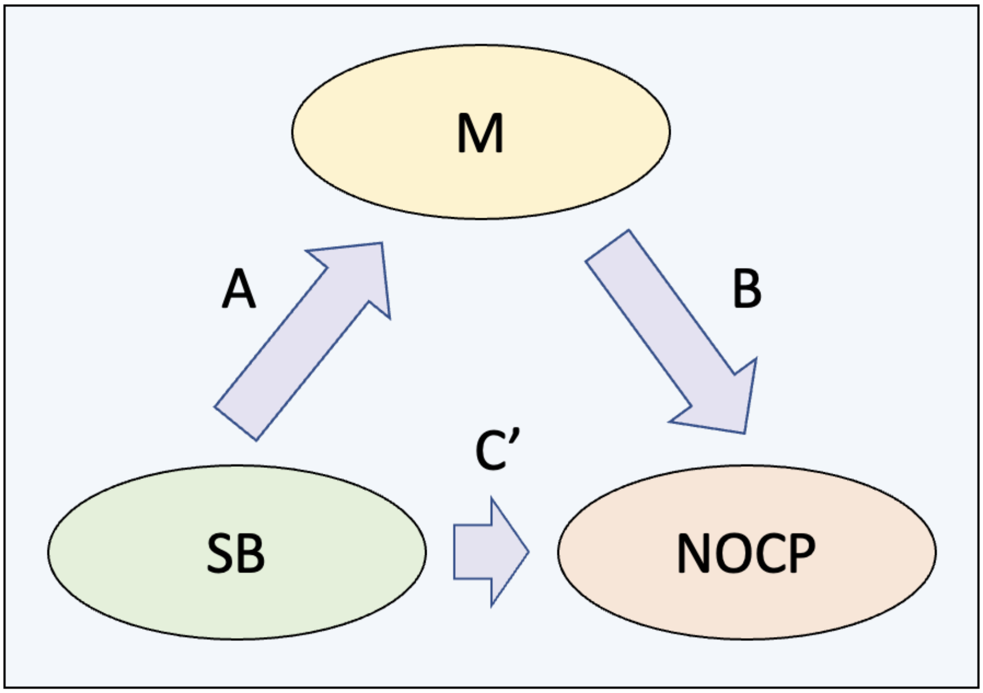

Appendix Figure 4. Average predicted risk of new onset of chronic pain in participants with and without sedentary behavior stratified by sex, after including sex as an effect modifier in the model shown in the multivariate analysis of Table 2.

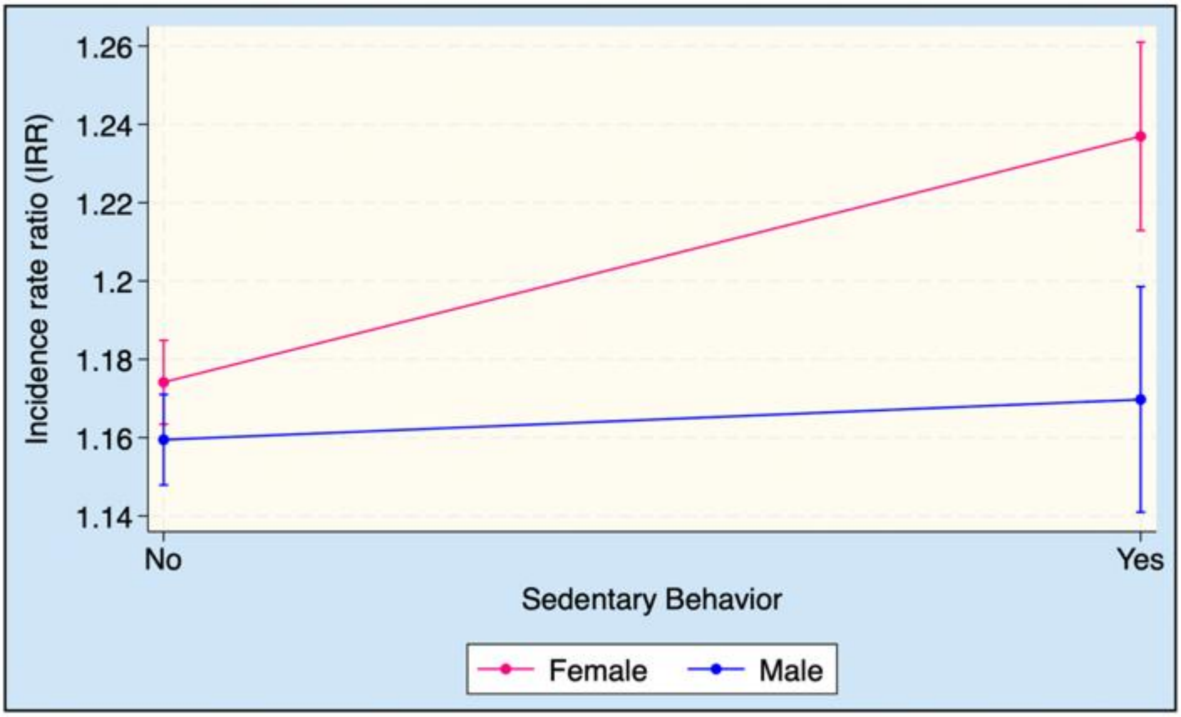

Supplement: Supplemental Material - Association Between Sedentary Behavior and the Development of New-Onset Chronic Pain in Older American Adults: Insights From the Health and Retirement Study [file sj-pdf-1-ahp-10.1177_08901171251412951.pdf]
